# Hantavirus Regulation of Type I Interferon Responses

**DOI:** 10.1155/2012/524024

**Published:** 2012-08-08

**Authors:** Valery Matthys, Erich R. Mackow

**Affiliations:** Department of Molecular Genetics & Microbiology, Stony Brook University, Stony Brook, NY 11794-5222, USA

## Abstract

Hantaviruses primarily infect human endothelial cells (ECs) and cause two highly lethal human diseases. Early addition of Type I interferon (IFN) to ECs blocks hantavirus replication and thus for hantaviruses to be pathogenic they need to prevent early interferon induction. PHV replication is blocked in human ECs, but not inhibited in IFN deficient VeroE6 cells and consistent with this, infecting ECs with PHV results in the early induction of IFN**β** and an array of interferon stimulated genes (ISGs). In contrast, ANDV, HTNV, NY-1V and TULV hantaviruses, inhibit early ISG induction and successfully replicate within human ECs. Hantavirus inhibition of IFN responses has been attributed to several viral proteins including regulation by the Gn proteins cytoplasmic tail (Gn-T). The Gn-T interferes with the formation of STING-TBK1-TRAF3 complexes required for IRF3 activation and IFN induction, while the PHV Gn-T fails to alter this complex or regulate IFN induction. These findings indicate that interfering with early IFN induction is necessary for hantaviruses to replicate in human ECs, and suggest that additional determinants are required for hantaviruses to be pathogenic. The mechanism by which Gn-Ts disrupt IFN signaling is likely to reveal potential therapeutic interventions and suggest protein targets for attenuating hantaviruses.

## 1. Introduction

### 1.1. Disease

Hantaviruses are present worldwide and responsible for two diseases: hemorrhagic fever with renal syndrome (HFRS) and hantavirus pulmonary syndrome (HPS). HFRS is primarily present in Eurasia and caused by several hantaviruses including Hantaan virus (HTNV), Seoul virus (SEOV), Puumala virus (PUUV) and Dobrava virus (DOBV) [1–3]. HFRS has a mortality rate ranging from 0.1–5% with causes of death including shock (75%), uremia (50%), pulmonary edema (15%), and central nervous system hemorrhage or encephalopathy (5%) [1–7]. In 1993, a discrete North American hantavirus (Sin Nombre virus, SNV) was found in the southwestern United States as the cause of a new highly lethal respiratory syndrome termed HPS [1, 2, 8–14]. HPS causing hantaviruses have since been found throughout the Americas [15–20]. Andes virus (ANDV) is a prototypic South American HPS causing hantavirus and the only hantavirus that is reportedly spread from person to person [21–24]. Although hantaviruses are predominantly pathogenic, Prospect Hill virus (PHV) and Tula virus (TULV) are hantaviruses which are not associated with human disease, and are referred to here as nonpathogenic, although it is unclear whether these viruses cause subclinical human infections [1, 2, 25–29].

In HFRS and HPS patients endothelial cells are ubiquitously infected throughout the body [5, 8, 13, 29, 30]. Hantavirus infection of the endothelium is nonlytic but results in prominent sequalae in the lungs and kidneys which contain vast endothelial cell beds. Consistent with altered fluid barrier functions of the infected endothelium, hantavirus diseases are characterized by increased vascular permeability, acute thrombocytopenia, hemorrhage, and pulmonary edema in the absence of endothelial cell lysis [5, 8, 13].

### 1.2. Transmission

Hantaviruses belong to the *Bunyaviridae* family and are the only members of the family transmitted to humans by discrete small mammal hosts [2, 14]. Hantavirus host specificity and the geographical host range distribution determine the potential for human HPS or HFRS diseases in worldwide populations [1, 2, 25, 31, 32]. Hantaviruses have coevolved with their small mammal hosts, persistently infecting their natural hosts in the absence of disease [32]. Infected hosts secrete hantavirus for prolonged periods of time and host-to-host transmission occurs through biting and virus excretion [1, 2, 13, 33]. Although hosts show no clinical manifestations of disease, it is unclear how hantaviruses evade host immune responses in order to establish viral persistence. 

### 1.3. Genome and Structure

Hantaviruses are enveloped negative-stranded RNA viruses ~100 nm in diameter with a spherical shape and a highly structured, grid-like surface [1, 2, 8]. Hantavirus genomes consist of three segments: small (S), medium (M) and large (L) [14]. The L segment encodes a single 220 kDa RNA-dependent RNA polymerase (Pol) which is highly conserved among hantaviruses but expressed at low levels in infected cells [1, 14]. S segment encodes the nucleocapsid (N) protein which is the most abundantly expressed hantavirus protein and the major antigenic determinant present in infected cells. N protein coats viral RNA and plays a role in virion assembly [14, 34, 35]. The S segment in TULV and PUUV contains an alternate internal ORF that encodes a short 90-amino-acid nonstructural protein (NSs) [25, 26, 36]. The NSs of TULV is suggested to play a role in IFN regulation; however, NSs is either truncated or not present in other hantaviruses [36, 37].

The M segment encodes a single precursor protein that is cotranslationally cleaved into two glycoproteins Gn (N-terminus) and Gc (C-terminus) presumably by cellular signal peptidases [14, 38]. Cleavage occurs after a universally conserved WAASA motif forming Gn and Gc glycoproteins that are trafficked and localized to the ER/cis-Golgi [39–42]. Gn and Gc are type I integral membrane proteins with their N-termini in the lumen of the ER and cytoplasmic C-termini [39, 41, 43, 44]. The cytoplasmic tail of Gc is only 9 amino acids long and contains a putative ER retention signal [45]. Gn contains a predicted signal sequence, several potential transmembrane domains, a double hydrophobic anchor sequence, predicted RING and zinc-finger domains, and a 142-amino-acid-long cytoplasmic tail (Gn-T) [39, 43, 45–50]. Virions are formed by budding into the lumen of the Golgi and exit the cell consistent with a secretory process [1, 14, 39, 43, 46].

The Gn-T of pathogenic hantaviruses has been shown to block the induction of IFN by upstream activators RIG-I and TBK1, but not IRF3-5D. The Gn-T of nonpathogenic PHV lacks the ability to regulate cellular IFN responses and may actually enhance pathway activation. In contrast, the Gn-T of nonpathogenic TULV regulates RIG-I- and TBK1-directed IFN induction similar to pathogenic strains [49–51]. The Gn-T of pathogenic hantaviruses harbor an ITAM motif and a C-terminal degron domain [47, 48, 52]. PHV and TULV Gn-Ts lack degron motifs and are stably expressed. Reciprocal changes between NY-1V and PHV have identified 4 residues that direct NY-1V Gn-T degradation [52]. Although degrons have been suggested to be present in PUUV, TULV and PHV [53], increased degradation of Gn-Ts from pathogenic NY-1V, SNV, ANDV, and HTNV, compared to PHV and TULV, have been reported [47, 51, 52]. The role of the degron remains unclear since the stably expressed Gn-T of TULV still regulates IFN induction and this suggests that the degron is not required for IFN regulation [51]. 

## 2. Hantaviruses and Type I Interferon Responses

### 2.1. Interferon

**Figure 1:**
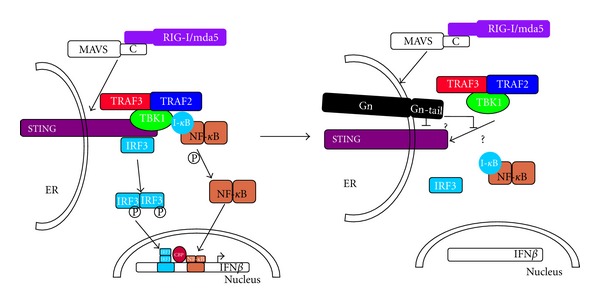
Potential model of hantavirus Gn-T disruption of STING-TBK1-IRF3 complex formation. Normally RIG-I/Mda5 recognition of viral RNA activates mitochondrial MAVS resulting in the downstream activation, phosphorylation, and dimerization of ER-resident STING [59–62, 65, 80]. STING is a scaffolding protein that binds TBK1 complexes through its C-terminal cytoplasmic domain [60, 61, 65], and STING-recruited TBK1 phosphorylates IRF3 and I*κ*B. This activates NF-*κ*B, permits IRF3 dimerization, and results in nuclear translocation of both IRF3 and NF-*κ*B which are both required for IFN*β* transcription. Expression of the NY-1V, ANDV, or TULV Gn-T inhibits RIG-I- and TBK1-directed IFN*β* transcription but has no effect on activated IRF3 [49–51]. Gn-T expression disrupts TBK1 binding to TRAF3 and acts at the level of STING-TBK1 complex formation to inhibit IRF3 and NF-*κ*B activation [49–51]. The specific interactions of the Gn-T with STING and TBK1 complexes that inhibit downstream pathway activation remain to be defined.

Type I IFNs (IFN*α*/*β*) are cytokines that are induced and secreted in response to viral infection and play a critical role in regulating viral replication [54, 55]. Many viruses regulate IFN induction in order to successfully replicate in cells [54]. Viral dsRNA or RNA elements are recognized by Toll-like receptors or intracellular RNA helicases that direct host cell signaling cascades leading to the induction of IFN*α*/*β* [56]. Intracellularly, the retinoic acid-inducible gene I (RIG-I) and melanoma-associated gene 5 (Mda5) function as cytoplasmic sensors of discrete types of viral RNA [57, 58]. RNA binding activates RIG-I and Mda5 and exposes tandem caspase activation and recruitment domains (CARDs). CARDs direct interactions with the mitochondrially located adaptor protein MAVS (also known as IPS-1/CARDIF/VISA), and further activate the assembly of downstream signaling complexes containing the ER-retained protein STING (stimulator of interferon genes) (also termed MITA/ERIS) [59–65] (Figure 1). STING a scaffolding protein that recruits TANK-binding kinase-1 (TBK1) and the interferon regulatory factor-3 (IRF-3) transcription factor along with a complex of TNF receptor-associated factors (TRAFs) required for IRF3 phosphorylation and NF-*κ*B activation [54, 58, 60, 62, 65–68]. TRAF3 forms homo- and heterotrimeric complexes with TRAF2, binds TBK1, and is required for IRF3 activation and IFN induction by virtually all signaling pathways [67, 69–72]. The transcriptional induction of IFN*β* requires both IRF3 and NF-*κ*B transcription factors to bind the IFN*β* promoter [54, 56, 66, 73]. TBK1 phosphorylates IRF3 resulting in the formation of phosphoIRF3 dimers that translocate into the nucleus (Figure 1). TBK1 also phosphorylates I*κ*B and this activates NF-*κ*B by permitting its nuclear translocation [73–76]. Once induced, IFN*β* is secreted by ECs and binds to IFN receptors (IFNAR) in an autocrine or paracrine manner triggering activation of Janus kinases (JAK) [77]. JAKs phosphorylate Signal Transduction and activators of transcription (STAT) factors, further activating downstream signaling pathways that lead to IFN induction and directing the production of many interferon-stimulated genes (ISGs) [78, 79]. The result is that this process is the production of a constellation of cellular ISGs that collectively inhibit various aspects of viral transcription and replication [54, 56, 66, 77, 78]. 

### 2.2. Endothelial Cell Responses during Hantavirus Infection

Several reports have shown that hantavirus replication can be blocked by pretreating cells with IFN*α*/*β* [49, 81–85]. Alff et al. demonstrated that pretreating ECs with IFN*α* blocks hantavirus replication, and inhibition is still observed when IFN*α* is added to ECs 6 to 12 hours after infection. Yet, the addition of IFN*α* 15 to 24 hours after infection had little effect on hantavirus replication [49]. This data supports the idea that pathogenic hantaviruses need to regulate the early induction of IFN in order to replicate successfully and is consistent with clinical data indicating that IFN treatment is only effective prophylactically or shortly after hantavirus infection [85, 86]. However, the timing of early IFN regulation may differ between specific hantaviruses depending on how rapidly they replicate within human endothelial cells [87, 88]. ANDV appears to regulate IFN-induced ISG56 protein expression for at least 12 hours with induction occurring by 24 hours. Subsequently hantaviruses cause a dramatic increase in ISG induction by 72 hours after infection [88, 89]. Another paper indicates that there is little if any increase in ISG induction following infection of epithelial A549 cells by a variety of hantaviruses [90].

While pathogenic hantaviruses infect and replicate in human ECs, studies have shown that replication of nonpathogenic PHV is severely restricted in ECs [49, 87]. In contrast to pathogenic HTNV, NY-1V, and ANDV hantaviruses, PHV was found to highly induce IFN and many ISGs in human ECs at early times after infection [87, 89]. Consistent with the near absence of PHV replication in ECs, S-segment RNA and nucleocapsid protein levels decreased 2 to 5 days after infection [87]. Conversely, pathogenic hantavirus titers increase from 1 to 5 days after EC infection and this occurred concomitantly with increased mRNA and nucleocapsid protein levels [49]. In contrast, pathogenic and nonpathogenic hantaviruses replicate to the same titers in Vero E6 cells that are deficient in IFN production and lack the type I IFN locus [91, 92]. 

DNA microarray analysis of hantavirus-infected ECs also revealed striking differences in the induction of ISGs by nonpathogenic PHV and pathogenic HTNV or New York-1 virus (NY-1V) [87]. PHV directs a high-level induction of many ISGs 1 day after infection while virtually no ISG responses were detected by the pathogenic strains NY-1V (HPS), ANDV (HPS), or HTNV (HFRS). Experiments using RT-PCR further demonstrated that PHV infection of ECs highly induced MxA and ISG56 (one day after infection), while pathogenic NY-1V or HTNV induced small MxA and ISG56 mRNA changes [49]. A separate study indicates that ANDV and PHV differ in their ability to regulate early ISG responses [89]. These observations were followed by studies contrasting PHV-induced IFN responses with pathogenic hantavirus-antagonized IFN responses following infection of human ECs [81, 87]. These reports suggest an increase in IFN production and ISG induction at early times after infection, which limits PHV replication [49, 89]. In contrast, pathogenic hantaviruses suppress the early induction of ISGs, thereby delaying the onset of early IFN responses and evading host defense mechanisms that would otherwise inhibit replication in human ECs [49, 87]. One possibility is that PHV lacks the ability to be a human pathogen since it actively induces early IFN responses and is unable to regulate early IFN induction within human ECs. However, IFN regulation is not the only determinant of hantavirus pathogenicity since nonpathogenic TULV regulates IFN responses and successfully replicates in human ECs [51]. These findings suggest that IFN regulation is necessary but not sufficient for hantaviruses to be human pathogens.

### 2.3. Viral Proteins Involved in IFN Regulation

#### 2.3.1. Nucleocapsid Protein (N Protein)

The viral proteins responsible for IFN regulation are reported to be the Gn cytoplasmic tail and the NSs proteins of some hantaviruses [37, 49–51, 89, 93]. Studies have demonstrated that N protein expression does not inhibit IFN or ISG induction by upstream IFN pathway activators RIG-I and TBK1 using luciferase reporter assays [49, 51, 94]. Although there are no studies indicating that N protein expression specifically regulates IFN induction, two studies report that N protein inhibits NF-*κ*B nuclear localization in response to TNF in A549 or 293T cells, yet NF-*κ*B activation is required for IFN promoter responses [95, 96]. N protein reportedly inhibits TNF-*α*-induced NF-*κ*B activation by preventing importin *α*4 binding to NF-*κ*B and its nuclear translocation [95, 96], while another study reported that N protein sequesters NF-*κ*B in the cytoplasm [89]. Although these reports suggest a broad regulation of NF-*κ*B by N protein, the involvement of N protein in NF-*κ*B regulation is contradicted by studies indicating that the N protein is unable to block IFN*β* or NF-*κ*B transcriptional responses directed by RIGI or TBK1 [49, 51, 94]. Thus N protein may specifically regulate a TNF-specific pathway of NF-*κ*B activation, but does not appear to be a ubiquitous NF-*κ*B inhibitor that blocks IFN induction. These conflicting results need to be resolved with common inducers, assays, and human ECs.

One report suggests that ANDV stimulates MxA expression in ECs 24 hours after infection and postulates that N protein forms a complex with MxA that interferes with S segment and N protein accumulation [81]. In a separate report, Levine et al. [94] suggest that ANDV and SNV modulate both the early IFN induction and the downstream JAK/STAT signaling pathway. ANDV and SNV were found to elicit a minimal or delayed expression of ISG56 and MxA in A549 and Huh7-TLR3 cells [94]. Expression of the SNV glycoprotein precursor acted as a potent inhibitor of IFN*β* and ISRE transcriptional activity, while expression of the SNV N protein was not observed to inhibit the induction of IFN, NF-*κ*B, or ISRE transcription. This study concluded that the early IFN responses are inhibited in SNV-infected cells due to the action of hantavirus glycoproteins while both the ANDV glycoprotein and N protein attenuate the effect of IFN at the JAK/STAT pathway [94]. However, these results do not explain why: (1) hantaviruses induce IFN and ISG responses in the presence of high levels of N protein; (2) all hantaviruses induce high levels of IFN and ISG at late times after infection; or (3) pathogenic hantavirus replication is insensitive to the late induction of IFN and occurs in the presence of high level ISG and MxA induction [87].

#### 2.3.2. Nonstructural Proteins (NSs)

Many bunyaviruses express nonstructural proteins (NSs) that have IFN regulating activity [93, 97–100]. The NSs protein of the Bunyamwera virus inhibits IRF3 and NF-*κ*B activation [100] while the NSs of Rift Valley fever virus (RVFV) interferes with IFN*β* mRNA transcription [97, 99]. A recent paper suggests that TULV and PUUV NSs proteins inhibit IFN*β* induction but the inhibition reported was only a 10–30% reduction in IFN responses, and it is unclear if this level of IFN reduction functionally reduces the antiviral effects of IFN [37, 93]. Further, a TULV strain expressing a truncated NSs was fully capable of replicating in IFN competent cells, although TULV strains expressing a full-length NSs reportedly survived for more passages. This finding suggested that NSs may have an overall effect on IFN-restricted growth [37]. Pathogenic hantaviruses ANDV, NY-1V, and HTNV either have truncated or nonexisting NSs proteins and it is unclear if NSs proteins contribute to IFN regulation by these pathogenic hantaviruses [37, 93]. 

#### 2.3.3. Glycoproteins

The hantavirus Gn protein is trafficked to the ER and contains a 142-residue-long cytoplasmic tail (Gn-T) that engages cytoplasmic viral and cellular proteins [1, 47–50, 52]. The Gn-T contains highly conserved domains that may have matrix protein-like functions for viral assembly at late times after infection, but which may also function in regulating early IFN responses [47–50, 52]. Several reports indicate that the pathogenic hantavirus Gn-T regulates IFN induction by blocking both IRF3 and NF-*κ*B activation [49, 50]. Gn-Ts from pathogenic NY-1V and ANDV, but not nonpathogenic PHV, inhibit IFN induction upstream of IRF3 activation at the level of the TBK1 complex [49, 50]. However, the Gn-T from nonpathogenic TULV also inhibits TBK1-directed NF-*κ*B and IRF3 activation indicating that IFN regulation is not limited to pathogenic hantaviruses. 

TBK1 is recruited to the C-terminus of the scaffolding protein, STING, which similar to Gn, is an ER-resident protein with a >100-residue-long cytoplasmic tail [60, 61, 64, 65]. The C-terminal 39 residues of STING bind TBK1 and are required to activate IRF3. Thus RIG-I/Mda5-MAVS activation of STING results in the recruitment of TBK1 complexes and the phosphorylation of IRF3 and IkB (Figure 1) [60, 62, 65]. The NY-1V Gn-T has been shown to co-IP TRAF3 but not TBK1 complexes and TRAF3 is a critical factor required for IRF3 phosphorylation and IFN*β* induction [50, 72, 101]. TRAF3 binds to the TRAF interacting motif (TIM) within MAVS through its C-terminal TRAF domain and may further link MAVS to STING activation events [67, 69, 71, 72, 102]. TRAF3 also binds to TBK1, linking upstream signaling responses of RIG-I/Mda5-MAVS-STING to the TBK1-directed activation of IRF3 and NF-*κ*B and transcription from IFN*β* and IFN response element (ISRE) containing promoters [67, 70, 71, 103, 104] (Figure 1). 

The Gn-T of NY-1V fails to bind TBK1 but co-IPs TRAF3 though its N-terminal domain, although residues required for Gn-T binding to TRAF3 have yet to be identified. Coexpressing the NY-1V Gn-T was also sufficient to prevent the formation of the TRAF3-TBK1 complexes consistent with the Gn-Ts ability to disrupt downstream signaling pathway activation and IFN induction. However, it is unclear whether co-IP or complex inhibition results from a direct interaction with TRAF3 or occurs via interaction with a complex assembled by STING that contains TRAF3 [50]. In contrast, the PHV Gn-T does not interact with TRAF3, is unable to block RIG-I or TBK1-directed IFN or ISRE transcriptional responses, and fails to inhibit TBK1-TRAF3 complex formation [49, 50]. These studies suggest that Gn-T interactions disrupt IFN-pathway-specific STING-TBK1-TRAF3 complexes [65], and suggest potential mechanisms for IFN regulation by a hantavirus protein. 

TULV is a serotypically distinct nonpathogenic hantavirus [25, 26] which, in contrast to PHV, successfully replicates in human ECs. This suggested its ability to regulate IFN responses like pathogenic hantaviruses [51, 105]. Prior studies comparing the innate immune response of HTNV and TULV cells suggested that ECs infected with TULV elicit a stronger IFN*β* response that induced MxA earlier than HTNV and increased HTNV replication [27]. However, the lower replication rate of TULV in ECs is contrary to recent studies where TULV-replicates successfully in ECs and reaches viral titers similar to levels obtained following pathogenic hantavirus infection [51, 105–107]. RT-PCR experiments measuring MxA and ISG56 mRNA levels in TULV-infected ECs further demonstrates that TULV regulates early IFN responses similar to pathogenic hantaviruses. Compared to PHV, TULV was found to suppress MxA and ISG56 responses 1 day after infection [51]. 

Analysis of the TULV Gn-T further showed its ability to inhibit TBK1-directed transcriptional responses from ISRE, IFN*β*, and *κ*B promoters similar to pathogenic hantaviruses [51]. Yet, unlike the pathogenic hantavirus Gn-Ts, the TULV Gn-T is unable to bind to TRAF3 [51]. In order to map the location of IFN regulation within Gn-Ts the ability of truncated expressed Gn-T proteins was investigated. The C-terminal 42 residues of the TULV Gn-T blocked TBK1- and RIG-I-directed ISRE and IFN transcriptional responses although it is unclear how the TULV Gn-T inhibits IFN induction in the absence of TRAF3-binding interactions [51]. However, recent data using degron mutants of NY-1V suggest that TRAF3 interactions are not required for the protein to regulate IFN responses, but are instead a function of degron interactions that may recruit TRAF3 or a TRAF3-associated E3 ligase complex to Gn-Ts. Thus far it is unclear what complex components and Gn-T residues are required for regulating IFN signaling pathway activation and transcriptional responses [51]. These findings demonstrate a need for studies of Gn-T interactions with discrete components of the STING-TBK1-IRF3 complex in order to elucidate this IFN regulatory mechanism (Figure 1). 

Several hypotheses have been proposed for how hantaviruses regulate cellular IFN responses. The C-terminal 42 amino acids of the Gn-T of pathogenic hantaviruses contain a degron domain that directs the ubiquitination and proteasomal degradation of Gn [52]. Binding of pathogenic hantavirus Gn-T to TRAF3, an E3 ubiquitin ligase, likely directs the ubiquitination and degradation of the pathogenic hantavirus Gn-T [103, 108] although another study of PUUV suggests that all Gn-Ts are ubiquitinated and degraded [53]. The state of TRAF3 ubiquitination regulates the formation of TBK1-directed transcriptional responses and it is possible that the interaction between the Gn-T of some hantaviruses and TRAF3 alters the ubiquitination state of TRAF3 and consequently inhibits IFN induction [51, 52]. However, if TRAF3 is not necessary for IFN regulation by the TULV Gn-T or degron deleted NY-1V Gn-T, it is also possible that hantavirus Gn-Ts commonly engage another component of the STING-TBK1-IRF3 complex [52]. Although interactions of the hantavirus Gn-tail with STING have yet to be investigated, it is interesting that the Gn-T from NY-1V, ANDV, and TULV block TBK1-directed ISRE, IFN*β*, and NF-*κ*B transcriptional responses directed by STING-TBK1 complex activation. Thus the ER-colocalized Gn-T may bind STING and interfere with STING dimerization, TBK1 recruitment to STING, or IRF3 recruitment to the STING-TBK1 complex [60, 61, 65] (Figure 1).

It is currently unknown which domains or residues within the cytoplasmic tail are required to inhibit IFN responses [51] and further studies are required to define hantavirus mechanisms of IFN regulation within human ECs. Identifying IFN regulatory elements is likely to permit the attenuation of pathogenic hantaviruses by generating hantaviruses that are unable to regulate IFN responses within human ECs but which are viable in IFN-deficient VeroE6 cells. 

#### 2.3.4. IFN Response as a Requirement for Pathogenesis

Pathogenic hantaviruses block early IFN responses but induce later high-level ISG responses (1–4 days after infection) [49, 87]. Despite the induction of many ISGs at late times after infection, hantaviruses replicate successfully in ECs, a finding that has been confirmed by showing that replication can be inhibited only if IFN is added less than 15 hours after infection [87]. Thus pathogenic hantaviruses have not only developed mechanisms to circumvent the early induction of IFN responses but they also become resistant to later IFN responses that might otherwise restrict hantavirus replication [52, 87, 89, 93, 95, 96]. The inability of PHV to regulate early IFN responses provides a rationale for its restriction in human ECs and explains at one level why PHV is incapable of being a human pathogen. 

Although IFN regulation is likely to be a requirement for hantaviruses to be pathogenic, TULV regulates IFN responses, replicates within ECs, and is not known to cause any human disease [51]. This demonstrates that although IFN regulation appears to be required for hantavirus replication, IFN regulation is not sufficient for hantaviruses to be human pathogens. In comparison with TULV and PHV, pathogenic hantaviruses block the function of *α*_v_ *β*_3_ integrin receptors which normally enhance fluid barrier functions of the endothelium [105, 109, 110]. Consistent with this, TULV infection of ECs does not alter EC permeability like pathogenic hantaviruses and this indicates that there are additional viral determinants of pathogenesis [105, 109, 110]. IFN regulation, integrin usage, and hantavirus-altered permeability responses are likely to be discrete determinants of hantavirus pathogenesis that may be required in concert to permit hantaviruses to be human pathogens. 

## 3. Conclusion

Several studies have established that pathogenic hantaviruses regulate the early induction of IFN responses by interfering with the IRF3 and NF-*κ*B signaling pathways, and the viral Gn-T is likely to regulate early IFN induction [49–51, 87, 89, 95, 96]. The Gn-T has been shown to inhibit RIG-I- and TBK1-directed IFN, ISRE and *κ*B transcriptional responses although the mechanism by which the Gn-T disrupts TBK1-directed IFN signaling responses remains to be defined [49, 50]. Viral proteins that regulate IFN responses, the timing of early IFN regulation, and IFN regulatory mechanisms may differ between hantaviruses. Determinants of IFN inhibition are located in the C-terminal 42 residues of the Gn-T but are likely modified by residues within the full-length tail, the degron in some proteins, and the presence of additional hantavirus proteins (N, NSs and Pol). Identifying residues necessary for IFN regulation will define elements that can be modified in order to attenuate hantaviruses and clarify mechanisms of IFN antagonism [51]. Although regulation of the early IFN response appears to be a crucial factor for the successful replication of hantaviruses in endothelial cells, it is clear that replication alone does not define a hantaviruses pathogenic potential. Thus replication in human endothelial cells is necessary but not sufficient for hantaviruses to be pathogenic and this suggests that additional pathogenic determinants are required for hantaviruses to be human pathogens [105–107, 109–111].
